# Leukoencephalopathy resolution after atypical mycobacterial treatment: a case report

**DOI:** 10.1186/s12883-015-0415-0

**Published:** 2015-09-02

**Authors:** Marcos C. B. Oliveira, Douglas Kazutoshi Sato, Herval R. Soares-Neto, Leandro T. Lucato, Dagoberto Callegaro, Ricardo Nitrini, Raphael S. S. Medeiros, Tatsuro Misu, Kazuo Fujihara, Luiz H. Castro

**Affiliations:** Department of Neurology, Faculdade de Medicina da Universidade de Sao Paulo, Av. Dr. Enéas de Carvalho Aguiar, 255, 5o andar, sala 5011, 05403-900 São Paulo, SP Brazil; Institute of Radiology, Faculdade de Medicina da Universidade de Sao Paulo, Av. Dr. Enéas de Carvalho Aguiar, 255, 5o andar, sala 5011, 05403-900 São Paulo, SP Brazil; Department of Pathology, Faculdade de Medicina da Universidade de Sao Paulo, Av. Dr. Enéas de Carvalho Aguiar, 255, 5o andar, sala 5011, 05403-900 São Paulo, SP Brazil; Departments of Neurology, Tohoku University School of Medicine, 2-1 Seiryo-machi, Aoba-ku, Sendai, Miyagi 980-8575 Japan; Multiple Sclerosis Therapeutics, Tohoku University School of Medicine, 2-1 Seiryo-machi, Aoba-ku, Sendai, Miyagi 980-8575 Japan

**Keywords:** Reversible encephalopathy syndrome, PRES, Mycobacteriosis, Leukoencephalopathy, Vasogenic edema, Aquaporin water-channel

## Abstract

**Background:**

Association of leukoencephalopathy and atypical mycobacteriosis has been rarely reported. We present a case that is relevant for its unusual presentation and because it may shed further light on the pathogenic mechanisms underlying reversible encephalopathies.

**Case report:**

We report the case of a Hispanic 64-year-old woman with cognitive decline and extensive leukoencephalopathy. Magnetic resonance imaging revealed white-matter lesions with increased water diffusivity, without blood–brain-barrier disruption. Brain biopsy showed tissue rarefaction with vacuolation, mild inflammation, few reactive astrocytes and decreased aquaporin water-channel expression in the lesions. Six months later, she was diagnosed with atypical mycobacterial pulmonary infection. Brain lesions resolved after antimycobacterial treatment.

**Conclusion:**

We hypothesize leukoencephalopathic changes and vasogenic edema were associated with decreased aquaporin expression. Further studies should clarify if reversible leukoencephalopathy has a causal relationship with decreased aquaporin expression and atypical mycobacterial infection, and mechanisms underlying leukoencephalopathy resolution after antimycobacterial treatment. This article may contribute to the understanding of pathogenic mechanisms underlying magnetic resonance imaging subcortical lesions and edema, which remain incompletely understood.

## Background

Reversible leukoencephalopathy is characterized by headaches, seizures, vomiting, and confusion, associated with extensive bilateral white-matter subcortical neuroimaging abnormalities, suggestive of vasogenic edema without infarction [1]. Most cases of reversible encephalopathy are referred to as posterior reversible encephalopathy syndrome (PRES), since white matter changes are more prominent in the posterior cerebral regions. Brainstem, cerebellum and other brain regions may also be affected [2]. PRES has been associated with severe hypertension (including eclampsia), renal dysfunction, infection, autoimune disease, and immunosuppressant drug use [1–7]. The hallmark of PRES is reversible cerebral edema due to blood–brain-barrier dysfunction [8]. Pathogenic mechanisms underlying magnetic resonance imaging (MRI) subcortical lesions and edema remains incompletely understood.

Association of brain edema and mycobacteria has previously been reported in the setting of *Mycobacterium tuberculosis* infection [9–15], and in one patient who presented with acute disseminated encephalomyelitis (ADEM) associated with *Mycobacterium intracellulare* meningitis, who responded to high dose steroid pulse therapy [16]. In all reported cases of mycobacterial infection associated with edema, underlying mechanisms involved demyelination and inflammation. Association of a non-demyelinating leukoencephalopathy and atypical mycobacteriosis has not, to our knowledge, been previously reported.

We report neuroimaging and brain histology features of a patient with extensive leukoencephalopathy with brain edema and absence of demyelination, that resolved after atypical mycobacterial pulmonary infection treatment.

## Case presentation

A Hispanic 64-year-old woman was admitted with headaches, vomiting and confusion. A month earlier, the patient presented with subacute new-onset headaches, nausea, vomiting, gait impairment, and anorexia. There was no history of fever, cough, abdominal pain, previous medical disease or immunosuppressant drug use. Blood pressure was normal. Physical examination was unremarkable. Neurologic examination showed an unsteady gait, without motor weakness or ataxia. Cognitive tests showed a Mini-Mental Status Exam (MMSE) score of 18/30, impaired attention, executive functions, verbal fluency and episodic memory (Table 1).

**Table 1:** Cognitive evaluations in a patient with reversible leukoencephalopathy before and after atypical mycobacteria treatment

	Initial assessment	Five months after initial assessment (no mycobacterial treatment)	Eighteen months after initial assessment (one year of mycobaterial treatment)
Mini Mental State Exam (MMSE)	18/30	18/30	22/30
Digit span (direct/indirect)	3 / 0	3 / 0	4 / 2
Short Memory Test with 10 items (incidental memory / immediate memory / learning)^*^	3 / 7 / 8	4 / 1 / 1	5 / 7 / 8
Delayed Memory Test with 10 items (after distraction) without / with hints^*^	6 (1) / 8	3 / 4 (3)	8 / 10
Verbal fluency (semantic / phonemic)	5 (animals) / 1 (letter P)	6 (animals) / 1 (letter P)	10 (animals) / 1 (letter P)
Clock-drawing test	Disexecutive: distortion of number placement (4 points)	Disexecutive: crowding numbers to one side (5 points)	More noticeable errors in hand/number placement (8 points)
Functional Activity Questionnaire (FAQ)		25/30	10/30

Patient’s education: 4 Years

Legend: ^*^Number of intrusions between brackets

**Fig. 1:**
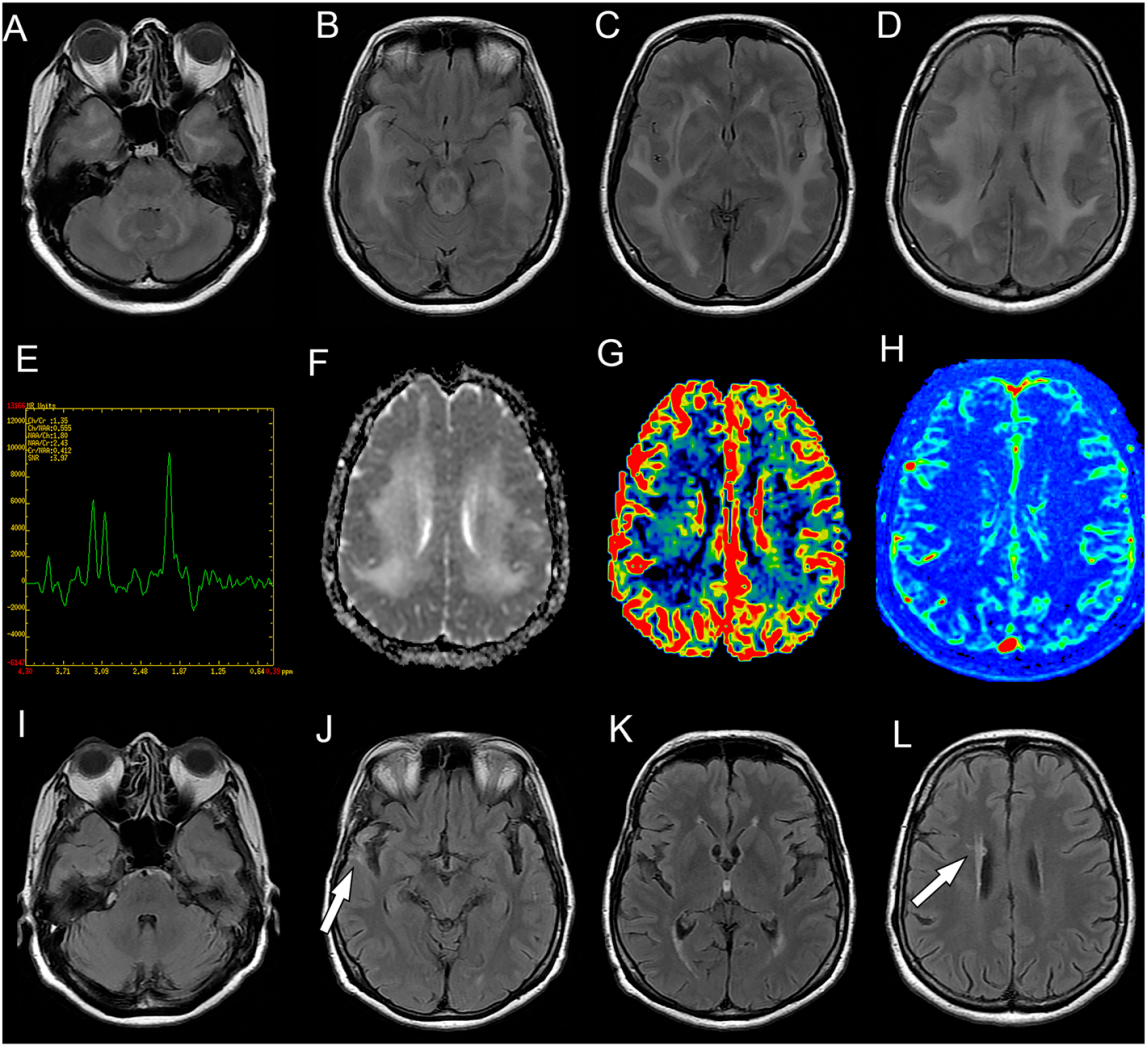
Serial brain magnetic resonance imaging studies of a patient with reversible leukoencephalopathy. Brain magnetic resonance imaging (MRI). Initial FLAIR images (**a**-**d**) show diffuse symmetrical confluent hyperintensities involving cerebral white matter, extending to the brainstem and cerebellar white matter. Note mass effect evidenced by sulci, fissure and ventricle effacement (more remarkable considering patient’s age - 64 years old). Corresponding white matter MR spectroscopy (**e**) (multivoxel, TE = 135 ms) demonstrates no definite metabolic changes. Apparent diffusion coefficients (ADC) map (**f**) demonstrates diffusion facilitation, signaling vasogenic edema. There was no contrast enhancement (not shown) or significant changes appreciated in color maps proportional to relative cerebral blood volume (rCBV) (**g**) obtained from a dynamic susceptibility contrast (DSC) perfusion (T2*) study. Color maps proportional to wash in rate (**h**) obtained from a dynamic contrast-enhanced (DCE) permeability (T1) sequence were also unremarkable. Images F-H are in the same level as D. After treatment for atypical mycobacteriosis, white matter changes disappeared, as shown in (FLAIR) images (**i**-**l**) obtained two years after the initial exam (arrows in J and L point to biopsy sites, partially characterized in these images)

**Fig. 2:**
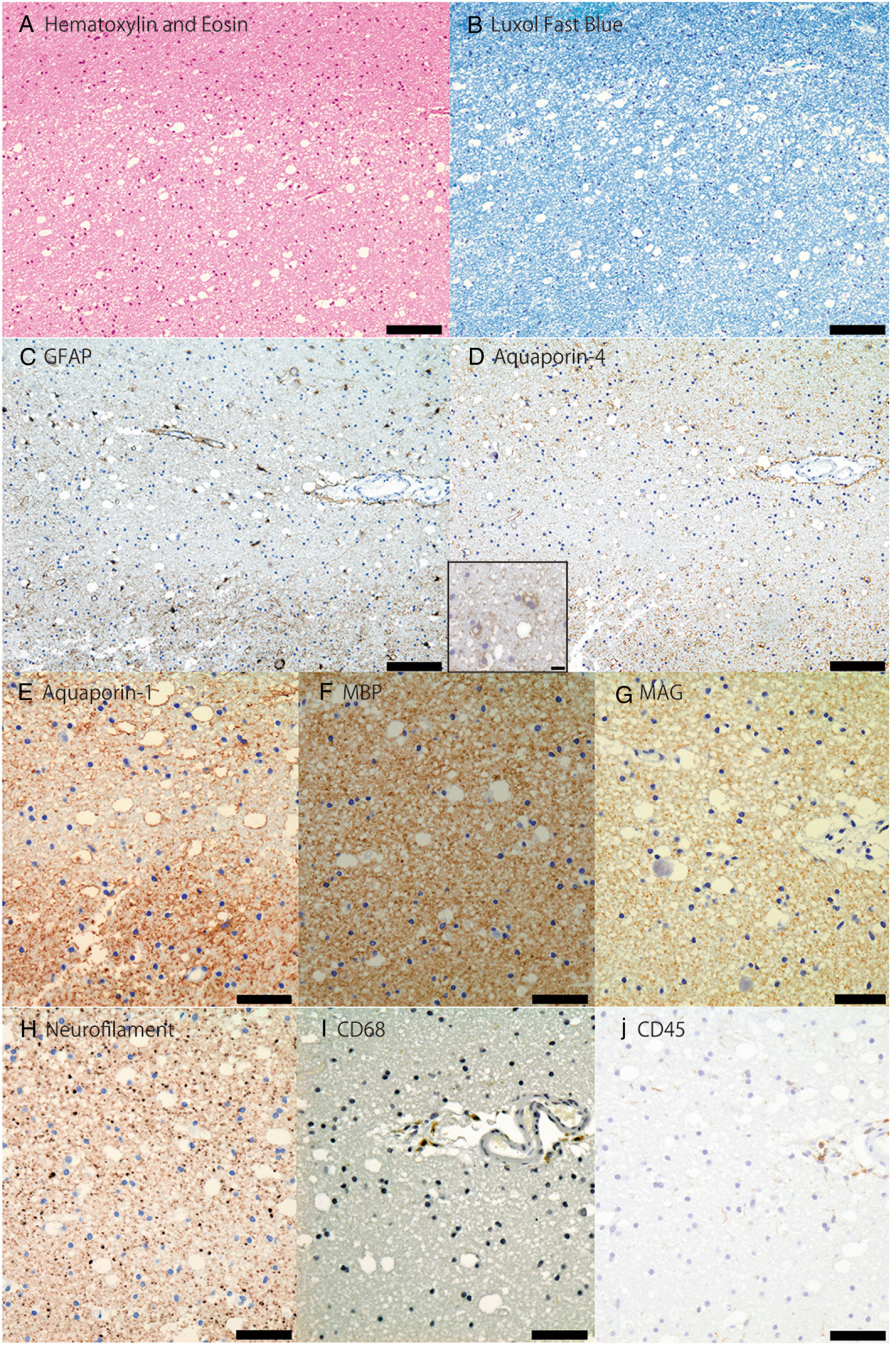
Brain biopsy results of a patient with reversible leukoencephalopathy. **a** to **j**: Brain biopsy results of the patient with reversible leukoencephalopathy prior to atypical mycobacteria treatment shows (**a**) mild tissue rarefaction with vacuolation, very sparse perivascular inflammatory infiltrates, and (**b**) no evidence of demyelination. **c**–**d** Presence of relatively few glial fibrillary acidic protein (GFAP) positive astrocytes with reduced aquaporin-4 expression in the lesion compared to the surrounding area. Scale bar = 100 μm. High magnification (400x) on D shows aquaporin-4 on the membrane of reactive astrocytes. Scale bar = 10 μm. **e** Aquaporin-1 expression is also reduced in the lesion, but in less extensively than aquaporin-4. **f**–**g** Myelin sheath is preserved with no loss of myelin basic protein (MBP) and myelin associated glycoprotein (MAG). **h** No signs of neuronal or axonal damage. **i**–**j** Few lymphocytes (CD45+) and macrophages (CD68+) are found in the perivascular space, while immunoglobulin and complement C9neo deposition are absent (not shown). Scale bar = 50 μm. (Magnification **a**–**d** = 100x; **e**–**j** = 200x)

Brain MRI disclosed diffuse and symmetric confluent nonenhancing white matter lesions, that were hyperintense in T2/FLAIR images (Fig. 1a to d). Corresponding apparent diffusion coefficients (ADC) maps suggested vasogenic edema (Fig. 1f). MRI angiography was unremarkable (not shown). Multivoxel spectroscopy, dynamic susceptibility contrast (DSC) perfusion (T2*) and dynamic contrast-enhanced (DCE) permeability (T1) did not disclose relevant abnormalities (Fig. 1e, g and h). Cerebrospinal fluid analysis showed a normal cell count (4 cells/mm3), protein (32 mg/dL) and glucose (80 mg/dL) levels, normal protein electrophoresis values, negative oligoclonal bands and polymerase chain reaction for infectious agents (including tuberculosis). Systemic evaluation was negative for cancer, autoimmune diseases (Anti-nuclear antibodies = negative, Anti-neutrophil cytoplasmic antibodies = negative), and infectious diseases. Thoracic computed tomography (CT) showed nonspecific patchy lung infiltrates. Blood laboratory tests were normal (i.e. Erythrocyte sedimentation rate = 2 mm; C-reactive protein = 1,3 mg/L; Leucocytes = 6,880/mm3). Electroencephalogram EEG showed mild diffuse slowing and brief bursts of diffuse delta waves. The patient underwent two brain biopsies that showed tissue rarefaction with vacuolation, very mild inflammatory cell and macrophage infiltrates, absence of demyelination, malignant cells or granulomas, and no signs of tissue infarction or hemorrhagic changes (Fig. 2a to j). Immunostaining showed scarce CD45+ lymphocytes and CD68+ macrophages, without axonal or myelin damage, with few reactive astrocytes and low aquaporin-4 staining in the lesion compared to the normal surrounding areas. Aquaporin-1 staining was also reduced in the lesion, less extensively than aquaporin-4.

The patient was treated initially with intravenous methylprednisolone (1 g/day for three days), followed by oral dexamethasone (10 mg/day) for six months. Clinical and neurologic status and brain MRI remained unchanged. Activities of daily living were impaired, with a Functional Activity Questionnaire (FAQ) score of 25 and MMSE score of 18. Whole body positron emission tomography-computed tomography obtained at this point revealed a hypermetabolic right pulmonary mass. Lesion histology showed granulomas containing *Mycobacterium abscessus*. The patient was treated with levofloxacin, clarithromycin and amycacin. Steroids were tapered and discontinued. A year later, cognitive functions and functional status were improved (MMSE = 21; FAQ score = 10) (Table 1), and brain MRI disclosed remarkable resolution of white matter changes (Fig. 1i–l).

## Conclusions

This report illustrates a case of leukoencephalopathy associated with atypical pulmonary mycobacteriosis. Although we cannot establish a cause-effect relationship of atypical mycobacteriosis and leukoencephalopathy, lack of central nervous system (CNS) granulomas and caseous necrosis, lack neurological worsening following steroid therapy, and improvement after antimycobacterial treatment suggest a remote effect of the lung infection, causing the CNS disorder.

Initial chest CT had initially shown nonspecific patchy lung infiltrates, that could, retrospectively, indicate early stage atypical mycobacterial infection. Additionally, the patient presented remarkable, albeit partial, neurological improvement after antimycobacterial treatment. There was no response to steroid therapy, brain pathology studies did not disclose inflammatory activity, and there was absence of intra-thecal antibody production, rendering the possibility of an adaptative immune mechanism (i.e. antibody or cell-mediated immune responses) extremely unlikely. Steroid therapy may have contributed to worsening of the mycobacterial lung infection.

Mycobacteria are known to be highly immunogenic: mycobacteria containing compounds are used in mouse models of ADEM and Multiple Sclerosis, through activation of adaptative immunity [17, 18]. Mycobacteria also activate the innate immune system, with production of cytokines and inflammation mediators, such as nitric oxide [19].

Mechanism of brain involvement in this case can be inferred from imaging and pathology findings of tissue edema with scarce reactive astrocytes, and reduced aquaporin-4 and aquaporin-1 expression in the lesion, compared to surrounding areas. Considering the lack of evidence of adaptative immune response in the brain, we speculate that activation of an innate-immune response in the lung either by the mycobacteria or through a host mediated response may have exerted a remote effect on aquaporin expression in the brain, leading to interstitial white matter edema. Alternatively, but less likely, antimycobacterial agents may have exerted a direct action reverting white matter lesions [20].

Few studies have evaluated pathological findings in reversible encephalopathies [1, 2, 8, 21], and some studies suggest the pathogenic role of cellular channel dysfunction. Reversible leukoencephalopathy has been described in few anti-aquaporin-4 antibody positive neuromyelitis optica (NMO) cases following immunotherapeutic interventions [3]. Lesion reversibility in these cases suggests that immune-mediated tissue destruction associated with blood–brain barrier (BBB) disruption may not be the main underlying mechanism.

It is conceivable that vasogenic edema noted on diffusion-weighted MRI sequences in reversible leukoencephalopathy cases represents parenchymal excess water content, caused by impaired water influx control, independent from BBB disruption. In our case, water influx control was probably impaired due to astrocyte aquaporin dysfunction, in a mechanism akin to that hypothesized in brain edema associated with NMO [3]. Additionally, experimentally induced acute hypertension in rabbits led to exogenous markers leakage in arterioles and capillaries through channels (often sigmoid-shaped) and cytoplasm and by transendothelial pinocytosis, causing brain-barrier disruption and edema [22]. These findings suggest impaired water channel function as a possible mechanism underlying reversible leukoencephalopathy.

We are not aware of previous non-demyelinating reversible leukoencephalopathy cases that improved after atypical mycobacteriosis treatment. We found vacuolated white matter lesions with paucity of reactive astrogliosis and decreased aquaporin water-channel expression. A causal relationship between mycobacteriosis and interstitial edema remains speculative. Alternatively, unexpected drug effects may have contributed to brain changes resolution. Elucidating pathogenic mechanisms underlying reversible leukoencephalopathies may lead to improved therapeutic strategies to treat this condition.

### Consent

Written informed consent was obtained from the patient for publication of this Case Report and any accompanying images. A copy of the written consent is available for review by the Editor-in-Chief of this journal.

## Abbreviations

ADEM: Acute disseminated encephalomyelitis
ADC: Apparent diffusion coefficients
BBB: Blood–brain barrier
CNS: Central nervous system
CT: Computed tomography
DCE: Dynamic contrast-enhanced
DSC: Dynamic susceptibility contrast
FLAIR: Fluid attenuation inversion recovery
FAQ: Functional Activity Questionnaire
MRI: Magnetic resonance imaging
MMSE: Mini-Mental Status Exam
NMO: Neuromyelitis optica
PRES: Posterior reversible encephalopathy syndrome
rCBV: Relative cerebral blood volume

## References

[1] Hinchey J, Chaves C, Appignani B, Breen J, Pao L, Wang A (1996). A reversible posterior leukoencephalopathy syndrome. N Engl J Med.

[2] Lee VH, Wijdicks EM, Manno EM, Rabinstein AA (2008). Clinical spectrum of reversible posterior leukoencephalopathy syndrome. Arch Neurol.

[3] Magaña SM, Matiello M, Pittock SJ, McKeon A, Lennon VA, Rabinstein AA (2009). Posterior reversible encephalopathy syndrome in neuromyelitis optica spectrum disorders. Neurology.

[4] Mak A, Chan BP, Yeh IB, Ho RC, Boey ML, Feng PH (2008). Neuropsychiatric lupus and reversible posterior leucoencephalopathy syndrome: a challenging clinical dilemma. Rheumatology.

[5] Bartynski WS, Boardman JF, Zeigler ZR, Shadduck RK, Lister J (2006). Posterior reversible encephalopathy syndrome in infection, sepsis, and shock. AJNR Am J Neuroradiol.

[6] Guerriero S, Ciraci L, Centoducati T, Pignatelli F, Lamargese V, Salvati A, et al. Bilateral Visual Loss as Presenting Symptom of Posterior Reversible Encephalopathy Syndrome in a Patient with HIV/Tuberculosis Coinfection: A Case Report. Case Rep Ophthalmol Med. 2012; doi: 10.1155/2012/850176 10.1155/2012/850176 PMC3518955 23243537

[7] Viehman JA, Khalil D, Barhoma C, Hanna RM (2013). Mycobacterium avium-intracellulare otomastoiditis in a young AIDS patient: case report and review of the literature. Hiv/Aids.

[8] Feske SK (2011). Posterior reversible encephalopathy syndrome: a review. Semin Neurol.

[9] Dastur DK, Udani PM (1966). The pathology and pathogenesis of tuberculous encephalopathy. Acta Neuropathol.

[10] Dastur DK (1986). The pathology and pathogenesis of tuberculous encephalopathy and myeloradiculopathy: a comparison with allergic encephalomyelitis. Childs Nerv Syst.

[11] Udani PM, Dastur DK (1970). Tuberculous encephalopathy with and without meningitis. Clinical features and pathological correlations. J Neurol Sci.

[12] Lammie GA, Hewlett RH, Schoeman JF, Donald PR (2007). Tuberculous encephalopathy: a reappraisal. Acta Neuropathol.

[13] Kim HJ, Shim KW, Lee MK, Park MS, Kim SH, Kim EY (2011). Tuberculous encephalopathy without meningitis: pathology and brain MRI findings. Eur Neurol.

[14] Char G, Morgan OS (2000). Tuberculous encephalopathy. A rare complication of pulmonary tuberculosis. West Indian Med J.

[15] Chetty KG, Kim RC, Mahutte CK (1997). Acute hemorrhagic leukoencephalitis during treatment for disseminated tuberculosis in a patient with AIDS. Int J Tuberc Lung Dis.

[16] Okada H, Yoshioka K (2010). Acute Disseminated Encephalomyelitis Associated with Meningitis due to Mycobacterium intracellulare. Inter Med.

[17] Wolf NA, Amouzegar TK, Swanborg RH (2007). Synergistic interaction between Toll-like receptor agonists is required for induction of experimental autoimmune encephalomyelitis in Lewis rats. J Neuroimmunol.

[18] Zorzella-Pezavento SF, Guerino CP, Chiuso-Minicucci F, França TG, Ishikawa LL, Masson AP (2013). BCG and BCG/DNAhsp65 Vaccinations Promote Protective Effects without Deleterious Consequences for Experimental Autoimmune Encephalomyelitis. Clin Dev Immunol.

[19] Lee J, Sandor M, Heninger E, Fabry Z (2010). Mycobacteria-induced suppression of autoimmunity in the central nervous system. J Neuroimmune Pharmacol.

[20] Dalhoff A, Shalit I (2003). Immunomodulatory effects of quinolones. Lancet Infect Dis.

[21] Chester EM, Agamanolis DP, Banker BQ, Victor M (1978). Hypertensive encephalopathy: a clinicopathologic study of 20 cases. Neurology.

[22] Hansson HA, Johansson B, Blomstrand C (1975). Ultrastructural studies on cerebrovascular permeability in acute hypertension. Acta Neuropathol.

